# *In Vivo*, Non-Invasive Characterization of Human Bone by Hybrid Broadband (600-1200 nm) Diffuse Optical and Correlation Spectroscopies

**DOI:** 10.1371/journal.pone.0168426

**Published:** 2016-12-20

**Authors:** Sanathana Konugolu Venkata Sekar, Marco Pagliazzi, Eugènia Negredo, Fabrizio Martelli, Andrea Farina, Alberto Dalla Mora, Claus Lindner, Parisa Farzam, Núria Pérez-Álvarez, Jordi Puig, Paola Taroni, Antonio Pifferi, Turgut Durduran

**Affiliations:** 1 Dipartimento di Fisica, Politecnico di Milano, Milano, Italy; 2 ICFO-Institut de Ciències Fotòniques, The Barcelona Institute of Science and Technology, Castelldefels, Barcelona, Spain; 3 Lluita contra la Sida Foundation, Germans Trias i Pujol University Hospital, Badalona, Spain. Universitat Autònoma de Barcelona, Barcelona, Spain; 4 Universitat de Vic-Universitat Central de Catalunya, Vic, Barcelona, Spain; 5 Dipartimento di Fisica e Astronomia, Università degli Studi di Firenze, Sesto Fiorentino, Firenze, Italy; 6 Consiglio Nazionale delle Ricerche - Istituto di Fotonica e Nanotecnologie, Milano, Italy; 7 Statistics and Operations Research Department, Universitat Politècnica de Catalunya, Barcelona, Spain; 8 Institució Catalana de Recerca i Estudis Avançats (ICREA), Barcelona, Spain; Medical Photonics Research Center, Hamamatsu University School of Medicine, JAPAN

## Abstract

Non-invasive *in vivo* diffuse optical characterization of human bone opens a new possibility of diagnosing bone related pathologies. We present an *in vivo* characterization performed on seventeen healthy subjects at six different superficial bone locations: radius distal, radius proximal, ulna distal, ulna proximal, trochanter and calcaneus. A tailored diffuse optical protocol for high penetration depth combined with the rather superficial nature of considered tissues ensured the effective probing of the bone tissue. Measurements were performed using a broadband system for Time-Resolved Diffuse Optical Spectroscopy (TRS) to assess mean absorption and reduced scattering spectra in the 600–1200 nm range and Diffuse Correlation Spectroscopy (DCS) to monitor microvascular blood flow. Significant variations among tissue constituents were found between different locations; with radius distal rich of collagen, suggesting it as a prominent location for bone related measurements, and calcaneus bone having highest blood flow among the body locations being considered. By using TRS and DCS together, we are able to probe the perfusion and oxygen consumption of the tissue without any contrast agents. Therefore, we predict that these methods will be able to evaluate the impairment of the oxygen metabolism of the bone at the point-of-care.

## Introduction

Diagnosis of bone related pathologies plays an important role in the ageing population of the world. Bone is subjected to continuous remodeling and damage due to daily activities, which in turn affect the tissue composition of bone [1]. Dual energy x-ray absorptiometry (DEXA) [2], the gold standard for diagnosing osteoporosis, measures the bone mineral density of trabecular bone, the porous (50% to 90%) bone that is commonly found at the extremities of long bones, of which radius distal and ulna distal are a few examples. Unfortunately, DEXA measures only bone mineral concentration and not collagen concentration [3]. Since collagen is the major constituent of connecting tissue present in bone, this limits the capabilities of DEXA in predicting the bone strength [3]. Moreover, the use of ionizing radiation highlights potential concerns on safety of repeated DEXA scans.

Another modality, ultrasound, mainly provides morphological and structural information, but fails in predicting the physiological status of the bone [4]. Other techniques, like x-ray scattering methods or Magnetic Resonance Imaging [5] (MRI), are under development to assess bone mineral, but all show limitations that prevent wide and effective application. *In vivo* Raman spectroscopy of the bone tissue [6,7] is a potential optical technique, but it is affected by a poor penetration depth. Importantly, along with techniques that assess bone mineral density, like DEXA, a complimentary technique for assessing organic material of bone like collagen, along with other tissue constituents (lipid, water) and hemodynamics parameters (oxy, deoxy-hemoglobin, blood flow index), can improve the predictability of bone related pathologies like osteoporosis.

Bone fibrous tissue consists of connective tissue (mostly collagen and hydroxyapatite), and cells. The concentration of collagen and other tissue constituents could be related to bone physiological conditions and to alterations induced due to pathological conditions [8]. It has been shown also that the blood flow decrease in vertebral cancellous bone is a marker for metabolic impairment associated with osteoporosis and osteopenia [9]. Collagen can be assessed non-invasively by diffuse optical techniques. High penetration depth along with the non-invasive nature of diffuse optical methods has found various applications in *in vivo* diagnostics: tissue characterization [10], hemodynamics monitoring [11,12,13], brain oximetry [11,14], and optical mammography [15,16,17,18] are few examples where the application of the technique has been explored deeply. Prior attempts to characterize bone tissue by diffuse optical techniques lack either the broadband nature [19,20] or number of subjects [21]. In reference to the problem of osteoporosis, preliminary *in vivo* results obtained by Pifferi et al., using a time resolved diffuse optical spectrometer [22] on calcaneus bone [21] of five volunteers suggested an interesting correlation of age with various tissue constituents (lipid, collagen, hemoglobin) and the scattering coefficient.

The optical properties in diffusive media can be assessed by time domain [23] (TD), continuous wave [24] (CW), or frequency domain [25] diffuse optics. In particular, high penetration depth and natural disentanglement of absorption from reduced scattering coefficient make TD an important tool for *in vivo* measurements on deep tissue, like bone. Importantly, a broadband TD system can extract the concentration of *in vivo* deep tissue constituents like collagen, lipid, water, hemodynamic parameters like oxy-, deoxy-hemoglobin, and oxygen saturation, making TD a promising technique to complement DEXA, which is insensitive to the organic part of bone tissue. Furthermore, Diffuse Correlation Spectroscopy, a non-invasive technique that allows the absolute estimate of the blood flow by optical means, at depths comparable with diffuse optical spectroscopy (few cm), has been shown in some studies to be able to measure the micro perfusion of bone [12,19].

In this work, we used both broadband Time-Resolved Diffuse Optical Spectroscopy (TRS) and Diffuse Correlation Spectroscopy (DCS) to measure the *in vivo* optical properties, tissue composition, and blood flow index in six different locations of seventeen subjects of age ranging between 25–50 years. Trabecular bone locations, where DEXA scans are usually taken, are sensitive to pathological changes at earlier stages. Thus, for our studies we chose these DEXA compatible locations, namely the distal and proximal extremities of ulna and radius in the arm (UD, UP, RD, RP), trochanter (T) near the femur of the hip in reflectance geometry, and calcaneus (C) in transmittance geometry. DEXA images were taken for all the locations except calcaneus. The penetration depth of the TD measurements was also estimated using theoretical means [26,27].

## Materials and Methods

### Instrumentation

**Fig 1:**
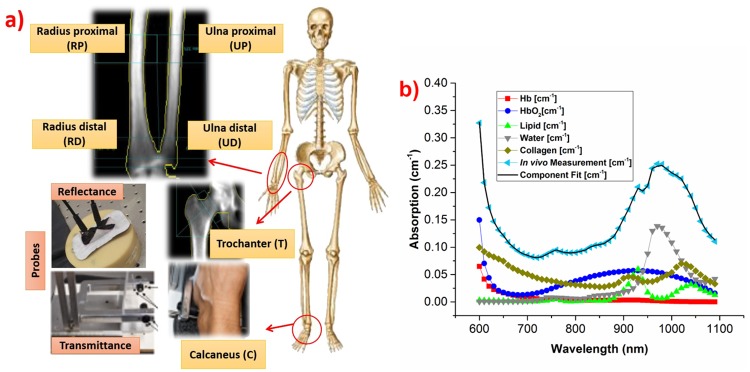
Schematic diagram of measurement protocol, and an example of tissue constituents fit. **(a) *In vivo* protocol locations**. Insets give a zoom view of bone located beneath the measurement region and show reflectance and transmittance probes. **(b) Example of *in vivo* absorption spectrum with weighted absorption spectra of tissue constituents** as obtained from the best fit to the experimental data.

In the TRS system, a supercontinuum fiber laser (SC450, Fianium, UK) powers the system with broadband (450–1750 nm) picosecond pulses. Source tuning is performed by the dispersion of light using an F2 glass Pellin Broca prism followed by an achromatic lens (f = 150 mm), which couples the light into the source fiber. To achieve broadband operation (600–1350 nm) while maintaining flat and high responsivity, two detectors are used, namely a Silicon Photomultiplier (SiPM, S10362-11-050C Hamamatsu, Japan, with home-made front-end electronics [28]) and an InGaAs Photomultiplier Tube (PMT, H10330A-45, Hamamatsu, Japan). In this work, we have limited our measurement range to 600–1200 nm, since the strong absorption of water prevented signal above 1200 nm. A drift and distortion control strategy enabled the real time operation in a clinical environment. More details on system construction and validation can be found elsewhere [29,30]. For the present study, TRS data were acquired every 10 nm and the maximum power on the tissue surface never exceeded 3 mW/mm^2^, which is within the maximum permissible exposure limits for the skin (as per BS EN 60825–1:2007, laser safety standard relevant to exposure on skin). The reflectance probe was made of PVC plastic with removable soft styrofoam at the skin contact end for sterilization before every measurement. For transmittance measurements at calcaneus, a customized U-shaped probe was designed to incorporate both TRS and DCS fibers. The probe is adjustable with a locking screw to accommodate wide variations in size and shape of calcaneus location. Insets in Fig 1(a) show the reflectance probe on a phantom, and the customized transmittance probe and its typical positioning at the calcaneus.

DCS measurements were carried with a custom-built setup, which was previously explained in detail [11]. A solid state, 785 nm CW laser with long coherence length (much longer than a typical photon path length) (DL785-120-SO, Crystalaser, USA) is coupled to a multimode optical fiber (core diameter of 200 μm, NA = 0.22) and attenuated to have less than 30 mW at the free end of the fiber. The ferrule at this end was held in a probe holder and placed perpendicularly on the surface of the body. A single mode fiber (core diameter 5.6 μm) was used to collect light on the same surface. The light collected this way was sent to a single photon avalanche photodiode in a four-channel array (AQ4C, Excelitas, Canada). The time series of detected photon signal is correlated over 7 s by a digital auto-correlator (Flex05, Correlator.com, USA).

### Subjects and Protocol

The entire protocol and instrumentation have been approved by the ethical committee (Ethic Committee from Hospital Universitari Germans Trias i Pujol, Badalona, Spain). Along with the health questionnaire recording the health history of the subject, an informed consent was duly signed before the measurement on the subjects; the study has been carried out in-line with Declaration of Helsinki principles. Seventeen healthy subjects spanning the age range of 20–50 years were recruited for this study. To track the general health status during the protocol period, blood pressure and pulse rate were monitored before and after the measurement. Fig 1(a) shows the DEXA compatible trabecular *in vivo* bone locations measured by the protocol. To estimate the superficial skin thickness and physiological condition of bone under study, skin caliper thickness and DEXA images of protocol defined locations (except calcaneus) were taken for all subjects.

First, TRS reflectance measurements were performed. Reflectance geometry with 25 mm as source-detector separation was used for radius distal (RD), radius proximal (RP), ulna distal (UD), ulna proximal (UP) and Trochanter (T) locations, as shown in Fig 1(a). To this end, the subject was asked to keep his/her arm in a comfortable position and the specially designed reflectance probe was handled by a trained person to maximized uniformity and reproducibility of the measurements. The last TRS measurement was done on the calcaneus (C), asking the subject to sit comfortably and keep his/her heel on the customized probe for transmittance measurements. The probe used for TRS measurements was also exploited to perform DCS in reflectance geometry at 25 mm source-detector separation for RD, RP, UD, UP, T locations and 32 mm for calcaneus location in the sequence mentioned above.

### Data Analysis

An analytical solution of the diffusion equation based on the extrapolated boundary condition [31] was used to extract absorption (*μ*_*a*_) and reduced scattering (*μ*_*s*_*’*) coefficients from acquired temporal curves. A homogeneous semi-infinite medium [31] and a finite slab were used for reflectance and transmittance geometries, respectively. Deeply penetrated photons were taken into account by fixing the fit range from 80% (0.80 times the maximum intensity) on the rising edge up to 1% (0.01 times the maximum intensity) on the falling edge of the temporal profile. More details on the fitting model are described elsewhere [31]. The scattering spectrum was approximated to the empirical power law derived from Mie theory [32]:

$$\mu \prime_{s}(\lambda )=a{\left(\lambda /\lambda_{0}\right)}^{-b}$$ (1)

where *λ* is the wavelength of light, *λ*_*0*_ = 600 nm, *a* and *b* are scattering coefficient, *a* = *μ'*_*s*_(*λ*_*0*_) is defined as scattering amplitude and it is related to the scatterer’s density whereas b is scattering power, characterizes the scatterer’s size. The fitting was performed in the range of 600–850 nm.

Key tissue constituents were quantified by fitting the *in vivo* absorption spectrum with a linear combination of the spectra of the major tissue constituents:

$$\mu_{a}(\lambda )=\underset{i}{\sum }C_{i}\varepsilon_{i}(\lambda )$$ (2)

where *c*_*i*_ is the concentration (free fit parameter) and *ε*_*i*_ signifies the specific absorption of the *i*^*th*^ component. In our bone measurements, we used 630–1100 nm range to fit spectral constituents. This is due to the lack of signal below 630 nm for few subjects and lack of oxy-, deoxy-hemoglobin spectrum above 1100 nm. Fig 1(b) shows the spectra of weighted tissue constituents (lipid, water, collagen, oxy-, deoxy-hemoglobin) corresponding to an *in vivo* spectrum measurement.

The DCS analysis is done using the correlation diffusion equation as model and the so-called Siegert relationship to relate the intensity autocorrelation functions that are measured to the modeled electric field auto-correlation functions [11]. A solution of the correlation diffusion equation [33,34] for a semi-infinite medium geometry [11] was used as a model to fit correlation data. A Brownian diffusion-like model of the mean squared displacement of the scatterers best fits *in vivo* data and allows us to extract a blood flow index (BFI). It has been previously shown that this approach is valid even in the measurement of the bone when using large source-detector separations as done in this study. We note that in order to obtain absolute values of the BFI, the absorption and scattering properties at 785 nm were obtained from the TRS analysis.

The variables recorded in the study were graphically described using means and box plots. The non-parametric Wilcoxon rank sum test was used to assess the intra-subject variation of tissue constituents at each location.

In order to have a first guess on the penetration depth of the TRS measurements, we applied a model which quantifies the mean value of the maximum penetration depth, 〈*Z*_max_〉, the average penetration depth, $\langle \bar{z}\rangle$, reached by detected photons as a function of the optical properties and photon travelling times [26,27]. The model used can work both in time and CW domain [27]. In diffusion conditions, the following relation holds $\langle \bar{z}\rangle \;=\;\langle Z_{\text{max}}\rangle /2$, and thus *<z*_*max*_*>* and $\langle \bar{z}\rangle$ are strictly related. In time domain techniques, depth is encoded in time. Maximum and average penetration depth of photons arriving at a specific time can be predicted using the reduced scattering coefficient and the arrival time of photons [27]. Thus, integrating 〈*Z*_max_〉 and $\langle \bar{z}\rangle$ of photons arriving at various times of the temporal profile and weighting with the photon intensity, we can provide the mean penetration depth of photons for given values of the reduced scattering coefficient. To this end, we simulated mean maximum and mean average penetration depth of photons by selecting for the integral of 〈*Z*_max_〉 and $\langle \bar{z}\rangle$, a time range in percent of the curve peak starting at 80% on the rising edge up to 1% in the falling edge of the temporal profile. For the calculation, we used the solution of the diffusion equation for the measured absorption and reduced scattering values at each wavelength and for a given bone location. The calculation of the penetration depth is done under the assumption that the medium investigated is homogeneous. Strictly, this is not true for the biological tissues under investigation that often show layered architectures. Therefore, the results presented in the next sections are subjected to this approximation [27]. For DCS involving CW light at 785 nm, the penetration depth was calculated using CW penetration depth model [27] for the optical properties at 785 nm. However, the effective depth sensitivity of DCS is often greater compared to CW near infrared spectroscopy when considering the contributions of differing amounts of moving scatterers, *i*.*e*. the red blood cell density, in different tissue layers [35]. The autocorrelation curve decay can be explained as a weighed sum of the number of scattering events in each region and the cumulative distance travelled by the scatterers. Once the superficial layer, with its presumably faster moving scatterers and with its relatively lower contribution to the number of scattering events, is traversed by the photon, the photon experiences a large number of scattering events and a weighed contribution from the bone and moving scatterers in the bone. Our previous results indicate that DCS is sensitive to bones that are several millimetres deep [19] and these considerations lead us to assume that the calculated values represent a conservative underestimation of the actual DCS sensitivity in depth.

## Results and Discussion

### Penetration Depth

**Fig 2:**
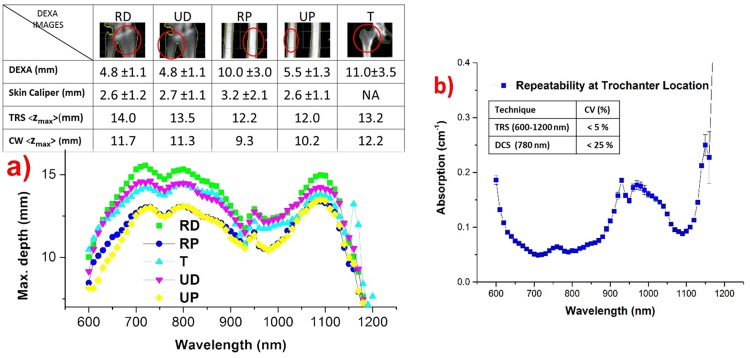
Results of penetration depth, superficial skin thickness calculations, and reproducibility measurements. **(a) Theoretical prediction of spectrally resolved penetration depth of TRS at each location**. DCS penetration is facilitated by operating at the wavelength of maximum penetration (785nm). The inset figure shows the DEXA scan and skin caliper measurements of superficial skin thickness (mean ± SD), and the mean maximum penetration depth for TRS averaged over 630–1100 nm range and CW at 785 nm. Proximal locations show relatively lower penetration depth than distal locations. **(b) Repeatability measurements** on the absorption spectrum (mean ± SD) at trochanter location, illustrating robustness and repeatability. The inset reports the CV for measurements performed with the two techniques (< 5% and 25% for TRS and DCS, respectively).

**Fig 3:**
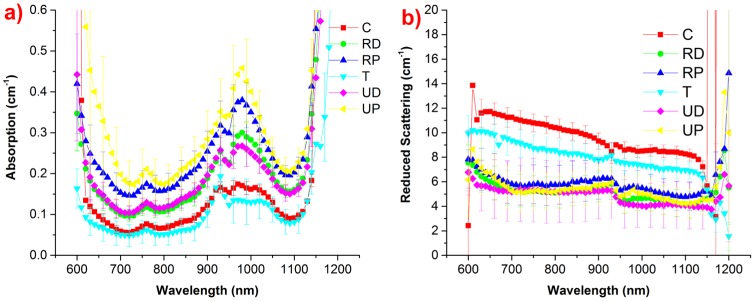
Average optical properties (absorption, reduced scattering co-efficient) at all 6 locations as measured by the bone protocol. **(a) Average absorption spectrum at each location**. A high absorption is seen in proximal locations, whereas trochanter and calcaneus with low average absorption. **(b) Average scattering spectrum of each location**. Distal and proximal locations show a similar scattering, while the calcaneus has highest scattering value.

The penetration depth of light as a function of wavelength at each location was obtained by using the theoretical model described in the sub-section on “Data Analysis”. Fig 2(a) shows the mean maximum depth for TRS as a function of wavelength, while in the inset displays the DEXA scan and skin caliper measurements of the superficial skin thickness, together with the TRS mean maximum depth averaged over spectral fit range 630–1100 nm and the CW mean maximum depth at 785 nm. For DCS—as a first (underestimated) guess—the CW mean maximum depth is displayed. We stress that the mean average depth is a factor of 2 lower than the data shown in the graph and in the inset for the maximum depth. Briefly, higher penetration depth is achieved in the presence of low absorption and/or low scattering. From Fig 2(a), distal locations were found to have larger penetration depth. Though trochanter has the least absorption (Fig 3(a)), high scattering (Fig 3(b)) prevents deep penetration of photons at the trochanter location. The superficial skin thickness was estimated from DEXA images and also from skin caliper readings. The reported DEXA thickness values are distances between the skin surface and the bone surface. The skin caliper is considered more reliable than the analysis of DEXA images for the estimate of skin thickness. This is due to the fact that the skin layer thickness is reduced by the pressure of the optical probe during measurements, which is more in line with the way the skin caliper measures the skin thickness. Fig 2(a) and the inset table clarify that over the entire spectral range and at every location the maximum penetration depth of light in *in vivo* measurements is larger than the actual depth of the bone. This confirms the validity of the protocol for the measurement of bone constituents. Importantly, the relatively small value of the average skin thickness on hand locations makes them to be more reliable locations for the extraction of bone constituents.

In DCS, sensitivity to deeper layers is facilitated with respect to CW spectroscopy due to differing amounts of moving scatterers in different tissues. Photons with long path lengths reach deep into the tissue as in TD. However, these photons that migrate deeply into the tissue undergo a higher number of scattering events with red blood cells and accumulate more photon momentum transfer along their paths, thus generating an amplified contribution to the earlier decay of the correlation function that in turn is analyzed in a way to increase DCS sensitivity to longer path length photons, in other words the earlier delay times [35,36]. This has been thoroughly tested in case of the adult human brain, where the underlying brain tissue with its higher blood flow contributes preferentially to the DCS signal compared to the superficial skull bone. In the case of our measurements, the situation is reversed, the photons that reach the bone contribute to the DCS signal only after many scattering events, implying that longer path length photons contribute more. In a previous study, it has been shown by compressing mechanically the superficial soft layer above the cancellous, manubrium bone [19], at levels of standard probe pressure, that the estimated BFI contribution is mainly from the bone.

### Repeatability and Repositioning Variations

Repeated measurements under perturbing environment were performed on trochanter location to demonstrate the reliable and robust nature of the measurements. To this end, the probe was placed by an untrained operator. Perturbations were introduced by repositioning and asking the subject to lean forwards, lean backwards, in stiff, and normal positions. Specifically, the subject took a position (*e*.*g*., leaning forwards), the probe was put into place, and four subsequent measurements were repeated. Then, the probe was taken away, the subject took the next position, and the measurement procedure was repeated until the last position. The results of the measurements are shown in Fig 2(b). The coefficient of variation (CV) of repeatability measurements is close to the limit of instrument’s inherent CV of 4% [29], proving the reliability and robustness of the protocol.

The same procedure was applied to evaluate the repeatability of DCS measurements of blood flow. An untrained operator was asked to replace the probe with the subject changing his position between measurements, as described before. Three measurements were obtained at each perturbation posture. The coefficient of variation was then estimated to be less than 25%, in reasonable agreement with what was expected for *in vivo* measurements, since BFI fluctuations in resting tissue tend to be few times higher than those of blood volume and oxygen saturation [11].

### Inter-location Variability of the Optical Properties

**Fig 4:**
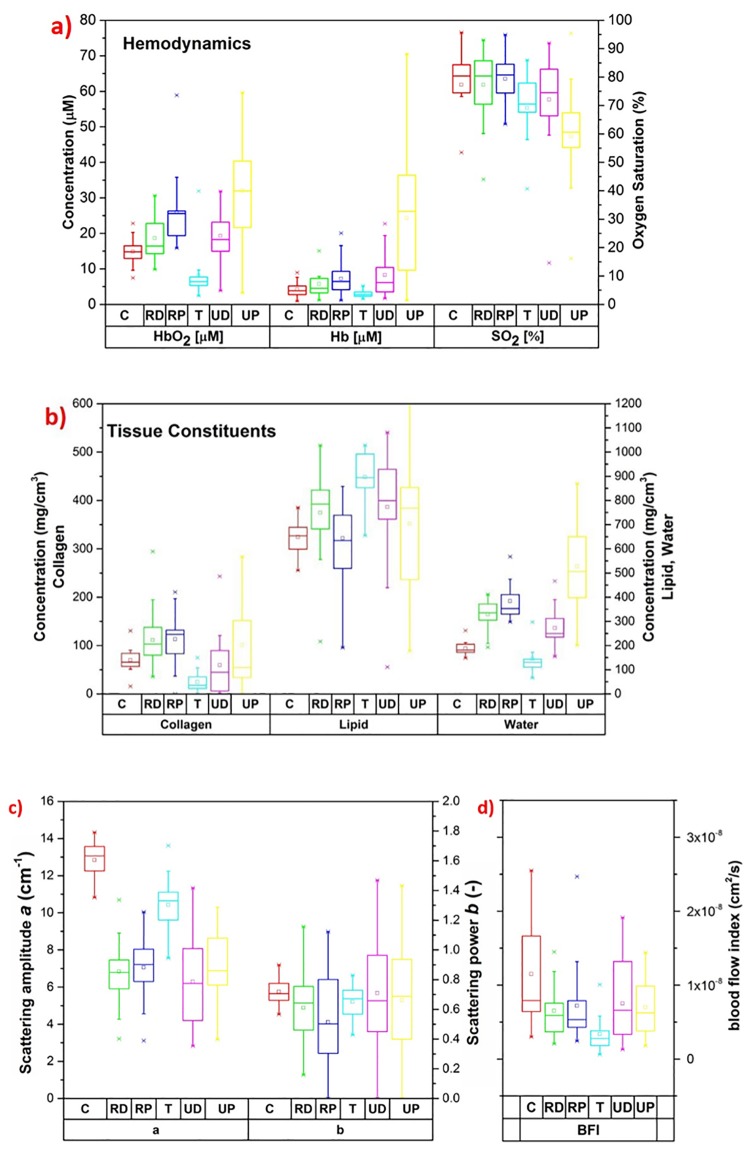
Box plots of results obtained by the diffuse optical studies on human bone. **(a) Box plots of hemodynamic parameters** at each location. **(b) Box plots of tissue constituents**. **(c) Box plots of scattering parameters** derived from Mie theory (Eq (1)). **(d) Box plots of the blood flow index**.

The visual analysis of the absorption spectra and reduced scattering spectra (Fig 3) provides qualitative trends on tissue composition and structure that are then quantified in the box plots of Fig 4.

A significant difference among the average absorption spectra of various protocol locations is evident from Fig 3(a). However, relatively closer values were found for the distal location pair (RD and UD), proximal location pair (RP and UP), and calcaneus-trochanter pair (C and T); indicating a similarity of absorption properties of these pairs. The highest average absorption is seen at the ulna proximal location, while the least absorption is observed at trochanter and calcaneus locations. Conversely, trochanter and calcaneus exhibit high average scattering spectrum. From Fig 3(b), a similar average scattering spectrum is observed for distal and proximal locations.

Long error bars on the average absorption spectrum of UP (Fig 3(a)) highlight a high inter-subject variability, which in turn is reflected in high inter-subject variation in estimated tissue constituents of UP (Fig 4). A non-parametric Wilcoxon rank sum test for intra-subject variation of tissue constituents showed that water and oxy-hemoglobin are significantly different (*p* < 0.05) at each location, whereas the other constituents showed significant differences for few combinations of locations. The calculated *p*-values are reported in Table 1. Overall, these results prove the heterogeneous nature of bone at distinct locations in the body, and to some extent even within the same location, as highlighted by the significant differences observed between distal and proximal locations of the same bone.

**Table 1:** p-values obtained by Wilcoxon rank sum test performed between the constituents of the measured locations. **Statistically significant values are underlined,** showing that most of the locations are significantly different from each other.

Wilcoxon test	C	RD	RP	T	UD	UP
p-values						
**RD**	**0.202**	**Hb**				
	***0*.*053***	**HbO2**				
	**0.782**	**SO2**				
	***0*.*004***	**Lipid**				
	***0*.*000***	**Water**				
	***0*.*017***	**Collagen**				
	***0*.*000***	**BFI**				
**RP**	***0*.*021***	**0.285**	**Hb**			
	***0*.*000***	***0*.*017***	**HbO2**			
	**0.605**	**0.730**	**SO2**			
	**0.890**	***0*.*042***	**Lipid**			
	***0*.*000***	**0.062**	**Water**			
	***0*.*007***	**0.558**	**Collagen**			
	***0*.*000***	**0.247**	**BFI**			
**T**	**0.121**	***0*.*017***	***0*.*002***	**Hb**		
	***0*.*000***	***0*.*000***	***0*.*000***	**HbO2**		
	**0.091**	**0.062**	***0*.*013***	**SO2**		
	***0*.*000***	***0*.*005***	***0*.*000***	**Lipid**		
	***0*.*008***	***0*.*000***	***0*.*000***	**Water**		
	***0*.*000***	***0*.*000***	***0*.*000***	**Collagen**		
	**0.148**	***0*.*000***	***0*.*000***	**BFI**		
**UD**	**0.109**	**0.513**	**0.836**	***0*.*009***	**Hb**	
	***0*.*011***	**0.469**	***0*.*050***	***0*.*000***	**HbO2**	
	**0.809**	**0.605**	**0.335**	**0.158**	**SO2**	
	***0*.*003***	**0.535**	***0*.*025***	**0.063**	**Lipid**	
	***0*.*000***	***0*.*010***	***0*.*000***	***0*.*000***	**Water**	
	**0.361**	***0*.*006***	***0*.*004***	***0*.*094***	**Collagen**	
	***0*.*002***	***0*.*068***	**0.281**	***0*.*000***	**BFI**	
**UP**	***0*.*000***	***0*.*000***	***0*.*001***	***0*.*000***	***0*.*001***	**Hb**
	***0*.*000***	***0*.*003***	**0.121**	***0*.*000***	***0*.*006***	**HbO2**
	***0*.*011***	***0*.*002***	***0*.*000***	***0*.*033***	***0*.*008***	**SO2**
	**0.098**	**0.809**	**0.352**	***0*.*021***	**0.449**	**Lipid**
	***0*.*000***	***0*.*001***	***0*.*021***	***0*.*000***	***0*.*000***	**Water**
	**0.931**	**0.370**	**0.343**	***0*.*022***	**0.342**	**Collagen**
	***0*.*000***	**0.543**	**0.625**	***0*.*000***	**0.244**	**BFI**

Oxygen saturation (SO_2_) varies between 70–90% for all locations, except UP that is characterized by the least SO_2_ (around 60% on average). Instead, maximum oxy- and deoxy hemoglobin are found in UP. The least total hemoglobin is seen at T location, in agreement with the adipose (less vascularized) nature of the superficial tissue that may affect the results more markedly at this location than elsewhere. Actually, an enhanced absorption at 930 nm (on the lipid peak) and the corresponding high lipid content evident from the box plot, combined with low collagen content, support the hypothesis of a high contribution of superficial tissue at trochanter location.

From Figs 3(a) and 4(b), the strong absorption of UP at 970–980 nm emphasizes a high water content of tissue, which proved to be maximum at this location. In general, proximal locations are found to have more water and hemoglobin (and less lipid) as compared to distal locations. Considering that blood and water are the major chromospheres responsible of the overall tissue absorption at short and long wavelengths, respectively, these results explain the high average absorption spectra of proximal locations shown in Fig 3(a).

From Fig 4(b), on average radius locations have more collagen than ulna locations. In particular, RD, combining high collagen values (that might be relevant to detect the patho-physiological condition of bone) with the highest penetration depth, seems a potentially interesting location for non-invasive optical studies of bone for diagnostic purposes.

Scattering amplitude *a* and power *b* were calculated by applying Eq (1) on *in vivo* reduced scattering spectra and a summary box plot is presented in Fig 4(c). A low intra-subject variation of the scattering power *b* indicates that the equivalent size of the scattering centers is similar at all locations. On the other end, calcaneus and trochanter have markedly higher scattering amplitude *a*, as already apparent from the clearly higher scattering spectra of C and T with respect to other locations (Fig 3(b)), in agreement with higher bone density of C and T (ref [37,38,39]). The observed values in hemodynamics and tissue constituents at calcaneus agrees with the earlier studies performed at this location [21,40].

### Inter-location Variability and Correlation of Blood Flow

A Wilcoxon rank sum test between pairs of locations shows that in most cases DCS data differ between locations. In particular, T location is significantly different with respect to RD, RP and UP. All the latter three locations are also found to be statistically different in terms of oxy- and deoxy-hemoglobin. Statistically significant variations between locations appear to reflect differences in most of the components concentrations measured by TRS. Fig 4(d) reports the box plot of BFI values obtained for the whole population divided by location. All the values are fairly dispersed and lie in the 7.5x10^-10^ to 5x10^-8^ cm^2^/s range. This is considerably larger than what previously reported from the sternum bone [19] that shows physiological BFI values in the range 2·10^−9^ to 1.4·10^−8^ cm^2^/s.

### Ideal location for Optical Probing of Bone *in vivo*

If an ideal location for diffuse optical studies of bone can be identified, it might profitably be exploited for optical diagnosis or monitoring of pathologies that affect globally bone properties, like osteoporosis. To identify an ideal location, we need to consider three parameters: the thickness of the superficial skin layer, the penetration depth of photons at the wavelength of operation, and the bone volume at the probed *in vivo* location. Fundamentally, minimum superficial skin thickness allows maximum probing of the underlying bone. However, we also need to consider that tissue optical properties play a significant role in the penetration of photons deep into tissues. In our current study, radius distal, ulna distal and ulna proximal locations have minimum superficial skin thickness (Fig 2(a)). However, as seen in Fig 3, the lower absorption of RD and comparable if not lower scattering compared to UP permits deeper penetration of photons at RD location. This also reflects in the penetration depth calculation displayed in Fig 2(a), where on average RD has higher maximum penetration depth than UP (14 mm *vs* 12 mm). UD has optical properties not markedly different from RD. However, Fig 2(a) shows that their combined contributions to the penetration depth make RD a better location than UD for deep bone probing. Importantly, DEXA images in Figs 1(a) and 2(a) reveal that ulna locations have low bone volume (thin bone) compared to radius bone locations. Indeed, collagen, a key constituent of connecting tissue of bone, is found to be highest in radius locations and minimal in ulna bone locations (Fig 4(b)), reconfirming the low bone volume nature of ulna locations. In summary, though RD, UD and UP have similar superficial skin thickness and penetration depth, the low bone volume of ulna locations has rejected them from being an ideal location for bone studies. Among RD and RP, RP is eliminated for high superficial skin thickness, high variability (SD) and low penetration depth. In contrast, RD has low superficial skill thickness, high penetration depth, and high bone volume along with low variability (SD), which make it an ideal and reliable location for diffuse optical studies of bone related pathologies.

### Limitations of the Study

The main limitation of the investigation is related to the heterogeneous nature of biological tissues and the homogeneity hypothesis assumed in the study. The main sources of heterogeneity can arise from the skin/epidermis and from the underlying tissue covering the bone (muscle, fat, cartilage). The bone tissue itself is locally heterogeneous with a trabecular structure filled with adipose tissue. This latter microstructure has no impact on the photon propagation since it was extensively proven—at least on Monte Carlo simulations—that the homogenization hypothesis is fully equivalent to the sub-mm heterogeneous microstructure [41]. The critical issues are related to the heterogeneous layers overlying the bone. To this respect, we can observe that both TRS and DCS are independent of contributions arising from the very superficial layer. Since the analysis is performed in both cases on the shape of the photon temporal or correlation function, this is not affected by thin absorbing layers (*e*.*g*., skin pigmentation, optical contact, hair), as observed—for instance—in measurements on red skin apples, where the retrieved absorption spectrum is not changed upon removing the skin [42]. For a similar reason, the contribution of the top few mm of tissues is negligible on the fitted TRS absorption. Indeed, the photon path length in superficial layers is rather constant upon increasing the photon traveling time. Thus, the fitted absorption—related to the slope of the photon temporal distribution—is feebly affected. Phantom measurements (source-detector separation = 2 cm, upper layer *μ*_*a*_ = ~0.15 cm^-1^ and *μ'*_*s*_ = ~15 cm^-1^) have demonstrated that a homogeneous fit retrieved the lower layer absorption within <15% discrepancy, as long as the thickness of the upper layer is ≤6 mm [43]. Also, comparing a two-layer model with a homogeneous model on the analysis of *in vivo* measurements on the forehead of 10 subjects, we obtained a good agreement in the estimate of *μ*_*a*_ of the lower layer (~10 mm deep) between the homogeneous fit and the two-layer fit [44].

A subtler distortion could arise from the spectral variation of the mean maximum penetration depth (Fig 2(a)), which could lead to a wrong weighing of the key components when tissue composition is estimated. The spectral change is not large (*e*.*g*., for RD in Fig 2(a), the maximum depth is within a range of 11–15 mm over the entire spectral range exploited for data fitting. Still, we have no experience on the possible impact on the retrieval of the tissue constituents.

More refined models—like two-layer geometries or even tomography—can be devised both for TRS and for DCS. Yet, these approaches often require more complex detection schemes, such as multi-fiber arrangements or significant dynamic range to accommodate multiple variables in the fit. If supported by clinical evidence on the usefulness of optical information for diagnosing bone related pathologies, the results presented here can represent a starting point, and lead to more advanced investigations that will take into account also the heterogeneous nature of the problem.

## Conclusion

A broadband diffuse optical characterization has been performed on various superficial *in vivo* bone locations. To the best of our knowledge, for the first time such a broadband study was carried out on multiple locations to quantify tissue composition from the absorption properties and derive information on microscopic structure from the scattering properties. In particular, all locations except trochanter showed significant content of collagen, a major organic component of bone, which may be of interest for diagnostic purposes. This was combined with the first extensive application of DCS to probe the bone physiology of healthy subjects in multiple body locations. The integration with TRS allows us to know the true optical properties of tissue and thus to assess the absolute value of the blood flow index in bone, paving the way for the future investigation of oxygen metabolism of the bone.

Our study performs the optical characterization of bone at locations that are traditionally considered for the assessment of bone mineral by DEXA scans. Investigating these locations with diffuse optics could open new methods for bone diagnosis. Main aim of this work was to demonstrate the feasibility of the diffuse optical characterization of the healthy bones and to establish baseline values and variability between locations and subjects. Furthermore, our goal was to assess the best location for diffuse optical monitoring. Taking into account penetration depth and actual superficial skin thickness, bone volume, radius distal location proves to be ideal for non-invasive bone probing. A key limitation of the study is the homogeneous model used for the analysis which could lead to contamination by the overlaying tissues or spectral distortions. Two-layer approaches are feasible for both TRS and DCS, yet at the cost of further complexity. Future studies will involve systematic *in vivo* measurements on subjects with a range of bone related pathologies, correlating various physiological parameters, pathological variations, and DEXA scan bone mineral density results with diffuse optical constituents for better understanding the feasibility of the above-mentioned diagnostic goal.

## Supporting Information

S1 Table Hemodynamic parameters. Estimated values of Hemodynamic parameters namely oxy-, deoxy-hemoglobin, oxygen saturation, blood flow index (BFI) at 6 protocol defined locations performed on seventeen healthy subjects. BFI was measured DCS whereas the other 3 parameters a were estimated by TRS technique.
(PDF)

S2 Table Tissue constituents. Estimated values of tissue constituents namely lipid, water, collagen at 6 protocol defined locations performed on seventeen healthy subjects.
(PDF)

S3 Table Scattering parameters. Estimated values of scattering coefficient namely scattering amplitude (a) and scattering power (*b*) at 6 protocol defined locations performed on seventeen healthy subjects.
(PDF)
