# Supplementary material for: In Vivo, Non-Invasive Characterization of Human Bone by Hybrid Broadband (600-1200 nm) Diffuse Optical and Correlation Spectroscopies
Source: PLoS One. 2016 Dec 20;11(12):e0168426. doi: 10.1371/journal.pone.0168426 (PMC5172608; doi:10.1371/journal.pone.0168426)
Supplement: S1 Table — Estimated values of Hemodynamic parameters namely oxy-, deoxy-hemoglobin, oxygen saturation, blood flow index (BFI) at 6 protocol defined locations performed on seventeen healthy subjects. BFI was measured DCS whereas the other 3 parameters a were estimated by TRS technique. (PDF) [file pone.0168426.s001.pdf]

S1-Table

Hemodynamics Parameters

C - Calcaneus **T -Trochanter**

RD - Radius Distal **RP - Radius Proximal**

UD - Ulna Distal **UP - Ulna Proximal**

Location	deoxy-hemoglobin (μM)	oxy-hemolgobin (μM)	oxygen saturation (%)	BFI (cm^2/s)
C	3.6	15.4	81.1	5.33E-09
C	4.6	14.4	75.8	3.00E-09
C	7.7	9.6	55.7	4.33E-09
C	4.5	12.3	73.1	8.85E-09
C	6.3	7.5	54.2	2.55E-08
C	3.3	15.7	82.7	2.15E-08
C	5.8	20.3	77.9	7.00E-09
C	2.7	22.8	89.3	2.55E-08
C	9.0	10.3	53.5	6.50E-09
C	4.0	16.1	80.3	9.90E-09
C	4.1	16.9	80.6	6.50E-09
C	1.9	15.1	89.0	9.31E-09
C	2.4	13.6	85.2	6.33E-09
C	0.9	19.6	95.7	1.18E-08
C	2.8	14.0	83.5	2.55E-08
C	3.7	14.1	79.3	7.00E-09
T	2.1	5.5	72.7	5.96E-09
T	3.5	2.4	41.4	2.95E-09
T	1.8	6.4	77.9	7.16E-09
T	4.8	3.3	40.7	2.08E-09
T	2.5	5.7	69.2	7.55E-09
T	2.4	5.1	68.2	1.02E-08
T	2.7	3.7	58.0	5.67E-09
T	1.9	9.4	83.2	3.69E-09
T	3.6	9.7	72.8	7.07E-09
T	3.1	6.4	67.6	1.45E-08
T	3.1	5.4	63.1	2.37E-09
T	2.3	5.4	70.5	1.05E-08
T	1.6	7.2	82.0	5.77E-09
T	3.0	6.8	69.4	4.65E-09
T	5.2	31.9	86.0	1.18E-08
T	4.0	15.7	79.5	5.90E-09
T	2.8	7.7	73.7	2.71E-09
RD	3.2	19.6	85.8	3.73E-09
RD	6.5	9.9	60.1	1.32E-08
RD	15.1	11.8	44.0	6.84E-09
RD	7.5	30.6	80.4	7.88E-09
RD	2.7	11.3	80.6	5.25E-09
RD	4.3	15.9	78.9	3.79E-09

RD	7.9	27.1	77.5	7.04E-09
RD	1.3	17.1	93.0	9.31E-09
RD	6.2	27.2	81.5	8.44E-09
RD	4.5	20.3	81.8	2.47E-08
RD	6.9	16.4	70.4	2.44E-09
RD	7.3	15.5	67.9	4.85E-09
RD	1.2	14.3	92.2	4.29E-09
RD	1.6	13.9	89.8	5.33E-09
RD	3.5	27.6	88.8	7.43E-09
RD	13.6	22.8	62.7	4.91E-09
RD	4.2	15.1	78.3	3.04E-09
UD	6.3	18.0	74.1	3.82E-09
UD	6.0	18.6	75.6	6.31E-10
UD	22.7	3.9	14.6	1.01E-09
UD	1.9	21.3	92.0	1.24E-09
UD	3.6	17.0	82.4	1.01E-08
UD	8.3	12.2	59.6	5.82E-09
UD	8.9	24.9	73.6	1.88E-09
UD	5.2	28.5	84.5	5.71E-09
UD	19.4	31.4	61.8	1.89E-09
UD	6.6	19.6	74.9	2.92E-09
UD	11.9	27.1	69.4	2.40E-09
UD	4.6	21.4	82.4	1.78E-09
UD	2.6	15.1	85.2	1.82E-09
UD	1.7	14.9	89.6	2.79E-09
UD	18.5	31.8	63.3	3.18E-09
UD	3.4	16.6	83.1	7.03E-09
UD	5.6	14.3	71.8	3.38E-09
RP	2.0	15.9	88.9	1.33E-08
RP	9.4	26.3	73.6	1.55E-09
RP	9.3	18.7	66.7	1.32E-08
RP	7.4	21.6	74.4	1.30E-09
RP	3.3	15.9	82.8	2.36E-09
RP	9.7	16.8	63.5	1.91E-08
RP	2.1	25.6	92.6	1.37E-08
RP	20.0	58.9	74.6	3.35E-09
RP	5.2	33.0	86.3	8.94E-09
RP	16.6	28.9	63.6	7.06E-09
RP	4.8	26.1	84.5	6.61E-09
RP	8.2	26.2	76.1	7.50E-09
RP	8.5	35.8	80.7	4.13E-09
RP	1.2	21.8	94.9	5.05E-09
RP	6.5	25.1	79.6	1.55E-08
RP	4.8	25.9	84.4	3.57E-09
RP	4.1	19.4	82.4	1.34E-09
UP	1.2	23.9	95.4	2.38E-09
UP	39.3	31.3	44.3	3.79E-09

UP	38.7	59.6	60.6	3.60E-09
UP	17.1	3.3	16.1	9.87E-09
UP	9.4	19.4	67.4	1.06E-08
UP	27.4	19.0	41.0	7.83E-09
UP	2.9	5.8	66.7	1.21E-08
UP	26.2	32.5	55.4	6.84E-09
UP	36.4	46.8	56.3	1.11E-08
UP	38.4	47.3	55.2	1.44E-08
UP	31.5	58.8	65.1	3.07E-09
UP	20.4	40.4	66.5	6.23E-09
UP	27.0	37.8	58.3	9.41E-09
UP	10.5	27.7	72.4	5.18E-09
UP	70.5	32.0	31.2	4.91E-09
UP	9.6	36.9	79.3	5.49E-09
UP	7.3	21.7	74.9	1.80E-09
