# Supplementary material for: In Vivo, Non-Invasive Characterization of Human Bone by Hybrid Broadband (600-1200 nm) Diffuse Optical and Correlation Spectroscopies
Source: PLoS One. 2016 Dec 20;11(12):e0168426. doi: 10.1371/journal.pone.0168426 (PMC5172608; doi:10.1371/journal.pone.0168426)
Supplement: S2 Table — Estimated values of tissue constituents namely lipid, water, collagen at 6 protocol defined locations performed on seventeen healthy subjects. (PDF) [file pone.0168426.s002.pdf]

S2-Table

Tissue Constituents

C - Calcaneus | **T -Trochanter**

RD - Radius Distal | **RP - Radius Proximal**

UD - Ulna Distal | **UP - Ulna Proximal**

Location	Lipid (mg/cm^3)	Water (mg/cm^3)	Collagen (mg/cm^3)
C	704.6	195.7	57.1
C	692.5	177.8	84.2
C	510.6	174.9	130.6
C	770.0	179.2	53.9
C	622.0	157.4	60.2
C	624.8	202.5	56.2
C	601.1	178.0	74.4
C	663.5	208.4	15.5
C	592.2	160.0	90.3
C	686.0	182.5	60.8
C	593.8	208.8	74.9
C	731.7	167.9	50.7
C	597.0	262.1	87.1
C	657.8	188.0	67.7
C	681.1	148.7	83.5
C	650.3	212.7	63.5
RD	606.0	335.2	118.7
RD	752.4	335.5	149.2
RD	216.8	374.4	294.2
RD	785.1	380.6	37.1
RD	800.4	209.2	49.9
RD	726.5	296.8	103.0
RD	842.8	318.8	93.6
RD	852.1	193.4	35.7
RD	814.9	399.1	194.8
RD	651.0	364.1	115.4
RD	947.4	319.7	80.1
RD	1027.4	327.1	51.5
RD	682.4	371.7	137.7
RD	556.1	291.4	144.3
RD	965.8	367.4	94.5
RD	687.0	411.2	111.4
RD	814.7	304.5	82.5
RP	662.1	323.9	123.2
RP	460.3	567.7	210.4
RP	415.5	442.3	169.0
RP	499.6	329.6	131.8
RP	749.2	321.1	36.8
RP	822.4	313.2	93.4
RP	615.0	335.1	130.8

RP	191.6	410.5	0.0
RP	738.7	458.8	123.2
RP	642.5	474.6	83.0
RP	1257.0	332.6	52.0
RP	728.1	401.6	130.6
RP	634.2	405.4	129.2
RP	561.1	352.7	197.0
RP	858.2	406.6	59.1
RP	518.6	345.1	111.9
RP	581.9	297.8	136.5
T	884.6	101.3	16.1
T	809.6	134.1	35.3
T	894.5	171.9	21.7
T	992.1	110.8	0.0
T	964.8	83.9	11.2
T	1005.1	66.4	13.9
T	852.7	130.2	33.9
T	955.2	114.4	0.0
T	655.0	297.1	74.6
T	853.5	131.6	21.2
T	664.0	118.3	52.3
T	825.3	143.3	17.6
T	1009.5	103.9	13.7
T	879.6	141.2	54.1
T	1028.2	249.9	0.0
T	1009.4	249.1	49.2
T	953.6	119.7	0.0
UD	798.9	316.7	43.8
UD	871.1	232.1	27.5
UD	111.3	312.4	243.1
UD	1007.7	154.8	0.0
UD	621.2	250.4	107.9
UD	870.6	276.2	68.8
UD	722.2	235.6	30.4
UD	928.6	317.3	89.6
UD	779.5	390.3	0.0
UD	1081.0	297.4	4.6
UD	994.6	235.1	0.0
UD	822.6	211.4	44.9
UD	750.6	249.7	91.0
UD	438.9	250.1	121.0
UD	977.2	466.1	6.2
UD	614.1	188.9	58.1
UD	743.5	246.4	70.7
UP	895.0	458.6	128.6
UP	351.7	609.8	151.8
UP	361.1	869.9	0.2

UP	718.4	248.2	225.2
UP	809.1	447.9	0.0
UP	607.0	534.8	123.5
UP	1006.1	201.8	54.5
UP	216.0	869.9	283.5
UP	473.0	735.9	260.5
UP	808.0	750.2	47.9
UP	1315.1	288.3	0.0
UP	1043.5	398.0	0.0
UP	768.1	506.7	50.8
UP	178.3	506.6	241.2
UP	853.8	650.1	34.0
UP	726.6	496.9	42.9
UP	826.9	386.2	67.1
