# Supplementary material for: In Vivo, Non-Invasive Characterization of Human Bone by Hybrid Broadband (600-1200 nm) Diffuse Optical and Correlation Spectroscopies
Source: PLoS One. 2016 Dec 20;11(12):e0168426. doi: 10.1371/journal.pone.0168426 (PMC5172608; doi:10.1371/journal.pone.0168426)
Supplement: S3 Table — Estimated values of scattering coefficient namely scattering amplitude (a) and scattering power (b) at 6 protocol defined locations performed on seventeen healthy subjects. (PDF) [file pone.0168426.s003.pdf]

S3-Table

Scattering Parameters

C - Calcaneus **T -Trochanter**

RD - Radius Distal **RP - Radius Proximal**

UD - Ulna Distal **UP - Ulna Proximal**

Location	scattering amplitue - a (cm^{-1})	scattering power - b
C	13.8	0.7
C	10.8	0.7
C	11.9	0.6
C	13.2	0.6
C	12.5	0.7
C	13.8	0.7
C	12.0	0.7
C	13.2	0.7
C	11.4	0.6
C	14.3	0.9
C	13.8	0.8
C	13.4	0.8
C	12.5	0.7
C	13.3	0.8
C	12.9	0.9
C	12.5	0.6
RD	6.3	0.8
RD	7.5	0.8
RD	7.2	1.2
RD	10.7	0.2
RD	4.7	0.6
RD	5.9	0.7
RD	8.1	0.7
RD	8.9	0.6
RD	6.8	0.8
RD	3.2	0.3
RD	10.0	0.6
RD	6.6	0.8
RD	5.6	0.4
RD	7.0	0.5
RD	4.3	0.5
RD	6.2	0.4
RD	7.3	0.7
RP	6.8	0.1
RP	3.1	0.3
RP	6.3	0.8
RP	6.5	1.1
RP	4.6	0.0
RP	8.0	0.3
RP	6.5	0.6

RP	10.0	0.9
RP	8.0	0.5
RP	4.9	0.5
RP	10.0	0.4
RP	7.2	0.9
RP	8.0	0.4
RP	7.5	0.3
RP	5.1	0.1
RP	8.4	0.8
RP	8.8	0.7
T	9.9	0.6
T	10.6	0.8
T	12.1	0.7
T	9.5	0.4
T	7.6	0.5
T	7.8	0.6
T	10.9	0.7
T	10.4	0.6
T	13.6	0.8
T	11.1	0.7
T	10.9	0.7
T	10.9	0.8
T	10.6	0.7
T	8.3	0.6
T	12.2	0.5
T	11.2	0.6
T	9.6	0.7
UD	2.8	0.2
UD	9.4	1.0
UD	4.2	1.5
UD	8.1	0.0
UD	9.3	0.6
UD	3.6	0.5
UD	4.2	1.0
UD	8.4	0.3
UD	5.8	0.6
UD	3.1	0.5
UD	6.2	1.2
UD	7.7	0.8
UD	6.9	0.4
UD	4.9	0.9
UD	3.9	1.0
UD	7.0	0.9
UD	11.3	0.7
UP	10.3	0.4
UP	6.9	0.9
UP	7.4	0.4

UP	6.0	1.2
UP	3.2	0.0
UP	6.7	0.7
UP	7.5	0.0
UP	5.9	0.4
UP	9.3	0.7
UP	6.1	1.0
UP	8.6	1.1
UP	6.6	0.3
UP	7.1	0.8
UP	6.7	0.5
UP	8.9	1.4
UP	4.8	0.7
UP	8.4	0.8
